# The Moderating Role of Autonomy Support Profiles in the Association Between Grit and Externalizing Problem Behavior Among Family-Bereaved Adolescents

**DOI:** 10.3389/fpsyg.2020.01578

**Published:** 2020-07-17

**Authors:** Lijuan Feng, Xiaoyu Lan

**Affiliations:** ^1^Student Mental Health Education and Counseling Center, Northwest Minzu University, Lanzhou, China; ^2^Faculty of Psychology, Beijing Normal University, Beijing, China

**Keywords:** externalizing problem behavior, perseverance of effort, consistency of interests, parental autonomy support, teacher autonomy support, family-bereaved adolescents

## Abstract

Research has consistently documented that the death of a close family member can disrupt a family’s functional equilibrium and has a deleterious effect on adolescents’ adaptation; however, little attention has been paid to behavioral adaptation of adolescents after a loss in a collective setting. Attempting to fill this research gap, the objectives of the current study are: (1) to identify autonomy support profiles based on two centered figures (parents and head teachers) and (2) to examine whether these emerging profiles may moderate the association between the two dimensions of grit (perseverance and consistency) and externalizing problem behavior in Chinese family-bereaved adolescents. A total of 763 family-bereaved adolescents aged from 13 to 18 years (60.3% girls; *M*_age_ = 15.74; *SD* = 1.53) were involved in the current study; they were asked to fill a battery of self-report questionnaires. A latent profile analysis revealed three autonomy support profiles: high parental autonomy support–high teacher autonomy support (HPHT; *n* = 598), high parental autonomy support–low teacher autonomy support (HPLT; *n* = 34), and low parental autonomy support–low teacher autonomy support (LPLT; *n* = 131). Moreover, results from linear regression analyses, after controlling for relevant bereavement variables, sociodemographics, and social desirability, showed that perseverance and consistency were negatively related to externalizing problem behavior for adolescents within the HPHL profile; nevertheless, the association between perseverance and externalizing problem behavior turned to be positive for adolescents within the HPLT profile. The current study sheds light on the variability of the association between grit and family-bereaved adolescents’ behavioral adaptation and further enriches the beneficial effect of autonomy support on adaptive functions in a collective cultural setting. The interplay between grit and autonomy support from parents and teachers has a significant influence on buffering externalizing problem behavior among family-bereaved adolescents.

## Introduction

The death of a close family member (e.g., a sibling or grandparent) can disrupt a family’s functional equilibrium and can have a profound effect on the entire family, affecting all surviving members and their relationships (Walsh and McGoldrick, 2013). For example, both siblings and grandparents contribute to an adolescent’s s role and identity consolidation (Hogan and Greenfield, 1991; Ens and BOND, 2005). However, a drastic change in these relationships can interfere with essential developmental processes, disturbing adolescents’ behavioral regulations. Although it is well-accepted that the death of a close family member places adolescents, in a Western cultural context, at risk for a number of adverse outcomes (Feigelman et al., 2017; Andriessen et al., 2018), little attention has been paid to behavioral adaptation of adolescents after a loss in a collective setting, such as in China.

Deeply rooted in Confucianism, the mainstream ideology in China attaches particular importance to family relations and dignity (Chang et al., 2003; Lan et al., 2019a). In this cultural context, the loss of a close family member often poses a traumatic and deleterious effect on adolescents’ behavioral adaptation. Nevertheless, several prominent features of Chinese culture make family-bereaved adolescents more vulnerable. To be specific, Chinese individuals are prone to suppressing their true sentiments due to cultural preference for modest personal expression, rather than straightforwardly expressing them as Westerners do (Soto et al., 2011; Pan and Liu, 2019). Likewise, Chinese individuals are less inclined than Westerners to proactively seek professional psychological counseling (Pan and Liu, 2019). Moreover, although adolescents who lose a close family member still attend school, grievers are not encouraged to visit their friends’ homes or be involved in any celebratory activities until at least 100 days after the loss because they are often assumed to be linked to bad luck (Chan and Mak, 2000). These salient features of Chinese society make it especially a meaningful context in which to investigate the possible risk and protective factors for behavioral adaptation of family-bereaved adolescents.

The delineation of risk and protective factors in this study emerges from the contextual resilience framework on adaptation after bereavement (Sandler et al., 2007). This framework posits that the death disrupts the equilibrium of relations between individuals and their environment and threatens their well-being and performance of developmentally appropriate roles. Moreover, this framework emphasizes the transactional interplay between the person and its environment on behavioral adaptation. To be specific, adaptation after a loss is shaped by not only by individual characteristics (e.g., individual’s beliefs about control over life events) and environmental (e.g., quality of relationship with parents and teachers) risk and protective factors but also the accumulation of multiple risk and protective factors that may proceed or follow the death. This framework has been successfully applied to empirical studies exploring risk and protective factors of adaptation in adolescents who suffer from a loss (e.g., Sasser et al., 2019).

To briefly summarize, the current study aims to investigate the possible protective roles of grit and perceived autonomy support (see the rationales of the selection of those variables later) against externalizing problem behavior among Chinese family-bereaved adolescents. In the following sections, we conduct a literature review of study variables, starting from the presentation of externalizing problem behavior.

### Externalizing Problem Behavior in Family-Bereaved Adolescents

Behavioral regulation in adolescence, as an essential aspect of psychosocial adjustment, has many consequences on the later development (Reitz et al., 2005; Van Heel et al., 2019). For instance, severe externalizing problem behavior negatively impacts adolescents in the close environment (e.g., parents, peers, and teachers) or society as a whole (Janssens et al., 2017). In the current study, we center on externalizing problem behavior, which is defined as rule-breaking and aggressive behavior (Achenbach, 1991). According to social information processing theory, individuals who are exposed to trauma (e.g., bereavement) may heighten the over-attribution of threatening intentions (e.g., misinterpretations of peers’ intentions) and become hypersensitive to social signals (Crick and Dodge, 1994; Abate et al., 2017; Lan and Moscardino, 2020). Within this perspective, consequences linked to family bereavement would thereby confer risk for later behavioral adaptation, such as exhibiting higher levels of externalizing problem behavior.

From an empirical research perspective, prior research has demonstrated that bereaved youths report higher levels of aggressive and delinquent behavior compared with those who have not experienced family bereavement (Kranzler et al., 1990; Draper and Hancock, 2011). Despite such findings, little research has been devoted to addressing the protective factors against externalizing problem behavior of family-bereaved adolescents in China. According to potential vulnerability reviewed earlier and the salience of family relationships in the context of Chinese culture, we aim to investigate the possible protective factors (e.g., grit) that may mitigate this harmful effect of family bereavement on externalizing problem behavior among Chinese family-bereaved adolescents.

### Grit

Grit is defined as the ability to strenuously and continuously pursue long-term goals, despite setbacks and challenges in the process (Duckworth et al., 2007; Duckworth and Quinn, 2009). We focus on the possible protective role of grit in this study based on the following theoretical and empirical considerations. As illustrated by the contextual resilience framework of adaptation after bereavement (Sandler et al., 2007), an individual’s belief about control over adverse life events is considered as one crucial protective factor for adolescents who suffer from a loss. In this regard, a gritty adolescent is more likely to show passion toward a specific goal and reframe the adverse events (e.g., family bereavement) more positively (Blalock et al., 2015; Lan and Radin, 2020; Lan et al., 2020).

Extant grit literature has predominantly addressed its linkage with academic performance (Credé et al., 2017; Datu et al., 2017; Muenks et al., 2017), but relatively little attention has been paid to the outcomes going beyond academia. In recent years, an emerging body of research has documented the positive effect of grit on various psychosocial functions in adolescence, such as better psychological well-being (Datu et al., 2018; Disabato et al., 2019), higher prosocial behavior (Lan et al., 2019a), and less problem behavior (Lan and Radin, 2020). Despite these research findings, research attention has primarily devoted to the association between an overall score of grit and adolescents’ adaptation. However, it is still unclear whether specific grit facets may mitigate the vulnerability distinctively.

Grit is represented by two dimensions: perseverance of effort (henceforth, “perseverance”) and consistency of interests (henceforth, “consistency”). Perseverance refers to the extent to which individuals can endure challenges while sustaining personal effort; by contrast, consistency reflects the degree to which individuals continually focus on achieving their long-term aspirations (Duckworth and Quinn, 2009; Lan and Radin, 2020). Research has underscored the differential effects of perseverance and consistency on adolescents’ adaptation (Datu et al., 2016; Disabato et al., 2019; Lan and Radin, 2020). For instance, Lan and Radin (2020) have shown that the ameliorating role of perseverance on externalizing problem behavior is more salient than consistency, particularly for adolescents with higher vulnerability. The authors explain their findings based on Chinese culture, highlighting diligence, determination, and perseverance when encountering possible difficulties.

Apart from the beneficial effect of grit on adolescents’ adaptation, a growing body of research has noted that there may be a “dark” side of grit (Anestis and Selby, 2015; Cui and Lan, 2020). For instance, research suggests that grit may contribute to a range of adverse outcomes, such as more frequent suicide attempts (Anestis and Selby, 2015). To be specific, when reporting greater persistence, individuals with higher levels of non-suicidal self-injury are more likely to report more frequent suicide attempts (Anestis and Selby, 2015). More recently, Cui and Lan (2020) have found that maternal harsh discipline is positively linked to aggressive behavior among adolescent boys reporting high levels of perseverance and consistency. The authors explain their findings in relation to the linkage between grit and impulsivity (Whiteside and Lynam, 2001), as being persistent and gritty in an unfavorable condition may be linked to more impulsivity and aggression. In this regard, these effects may be moderated by the environment that they are in (e.g., families and schools), and more research is needed to explore these conditional effects so as to understand better when grit is positively linked to psychosocial outcomes. Indeed, adolescents’ capacity to adequately cope with stress depends largely on the nature of the stress and on the social support resources to diminish or counter the effects linked to the stressor. In this study, we focus on the impact of perceived autonomy support.

### Perceived Autonomy Support

Perceived autonomy support involves an interpersonal style in which parents or teachers take the perspective of their children or students into account, present rationales for demands, acknowledge their feelings, and provide opportunities for choice and self-determination (Deci and Ryan, 2000). In this study, we center on the possible protective role of perceived autonomy support based on the following considerations. Informed by self-determination theory (SDT; Deci and Ryan, 2000), autonomy is regarded as one of the basic psychological needs that contribute to adaptive psychosocial functions. Such a psychological need is particularly highlighted in adolescence due to the increasing demand for autonomy-seeking during this period. Extant research based on SDT indicates that greater autonomous support is positively associated with both less stress incursion and more active coping with stressful events, which in turn add to resilience (Weinstein and Ryan, 2011). In this regard, autonomy support may facilitate adolescents’ integration of their bereavement experiences, which may enable adolescents to be more capable of regulating their behaviors.

Moreover, given the salience role of autonomous regulations, in this study, we focus on two significant sources of autonomy support during adolescence: parents and head teachers. This is in accordance with the socioecological framework (Bronfenbrenner, 1979), which emphasizes that parents and teachers are assumed to be the most proximal socialization agents in adolescent development. As a note, we focus on the role of head teachers in this study because head teachers usually instruct the same group of students in the whole education phase and take the primary responsibility of the social, administrative, and academic activities of the class in the Chinese school system (Chen et al., 2003). In this sense, head teachers’ autonomy support may be more salient and available in terms of facilitating family-bereaved adolescents’ adaptive functions.

Although the beneficial effect of autonomy support on adaptive functions has been extensively documented, extensive studies adopt a variable-centered approach to address the association between perceived autonomy support and adolescents’ adaptation. Such an approach is albeit informative and valuable, based on the assumption that the sample is homogeneous concerning the investigated associations. For instance, a two-way interaction identified in linear regression is assumed to apply to all adolescents in the sample. However, subgroups of adolescents may exist in any sample, and these variables may operate very distinctly within each subgroup (Stanley et al., 2017; Cui and Lan, 2020). To address this limitation, we use a person-centered approach [i.e., latent profile analysis (LPA)] to explore autonomy support profiles. LPA relies on probabilities and fit statistics to identify the optimal number and nature of subgroups (Stanley et al., 2017). The latent variable indicates that those adolescents belonging to a specific profile have a higher probability of sharing similar characteristics compared with those belonging to other profiles.

Extant research has used a person-centered approach to explore autonomy support profiles, which may provide a solid rationale for the current study. For example, Guay et al. (2013), utilizing three significant sources (mothers, fathers, and teachers), have unraveled three distinct autonomy support patterns in adolescents: low levels of autonomy support on all sources, low levels of autonomy support from fathers–moderate levels of autonomy support from mothers and teachers, and moderate levels of autonomy support on all sources. These patterns demonstrate that adolescents in the second profile report equivalent autonomous regulations and perceived competence to those in the third profile. Moreover, in a sample of university students, Ratelle et al. (2013), adopting three significant sources (parents, friends, and the romantic partner), have found five autonomy support profiles: high levels of autonomy support on all sources, moderate levels of autonomy support on all sources, low levels of autonomy support from parents and friends–moderate levels of autonomy support on a romantic partner, low levels of autonomy support on all sources, and moderate levels of autonomy support on parents and romantic partner–low levels of autonomy support from friends. Subsequently, they claim that when all sources are perceived as highly autonomy-supportive, individuals exhibit the highest standards of subjective well-being.

### The Present Study

The goals of the current study are twofold. First, we rely on a person-centered approach to identify autonomy support profiles, according to parental autonomy support and teacher autonomy support in family-bereaved adolescents. Second, we examine the direct and interactive associations of grit (i.e., perseverance and consistency) and these emerging autonomy support profiles with externalizing problem behavior in family-bereaved adolescents.

With regard to the first research aim, given the exploratory nature of identifying these profiles, no specific hypothesis is made. Nevertheless, based on the literature reviewed earlier, we expect that there may be a profile highlighting high levels of autonomy support on all sources and one pattern that may be characterized by low levels of autonomy support on all sources. Likewise, the dimensions of parental autonomy support and teacher autonomy support can be differentiated, and both sources may co-occur to different degrees, thereby constituting different autonomy support profiles.

In terms of the second research aim, we anticipate that perseverance and consistency are negatively linked to externalizing problem behavior. Moreover, this association is moderated by emerging autonomy support profiles. To be specific, in a favorable condition (high levels of autonomy support on all sources), the association between perseverance/consistency and externalizing problem behavior is positive; in contrast, the corresponding association may be detrimental in an unfavorable condition (low levels of autonomy support on all sources).

Moreover, prior research has indicated that sociodemographic variables and relevant bereavement variables are potentially linked to externalizing problem behavior (Andriessen et al., 2018; Cui and Lan, 2020). Additionally, given that socially desirable responses may bias or underestimate self-report data (Podsakoff et al., 2003), we also control the levels of social desirability in the present research. Taken together, we regard age, sex, family socioeconomic status (SES), social desirability, the degree of possible trauma effect, and the duration of losing a close family member as potential covariates in this study.

## Materials and Methods

### Participants and Procedures

A total of 763 family-bereaved adolescents aged from 13 to 18 years (60.3% girls; *M*_age_ = 15.74; *SD* = 1.53) were involved in the current study. We adhered to convenience sampling in this study. Participants were recruited from public middle and high schools located in urban areas of north mainland China, relying on personal networks and school collaborations. In this study, “family-bereaved” refers to adolescents who have lost one or more close family members (i.e., grandparents or siblings) due to intentional (e.g., chronic physical disease or naturally occurring death) or unintentional death (e.g., accident or suicide). The average duration of losing a close family member was 4.57 years (*SD* = 2.97). As a note, we omitted 49 parent-bereaved adolescents and 150 adolescents whose parents divorced from the initial sample^1^. During data collection, participants were in grades 7, 8, 10, and 11 in schools. We excluded 9th and 12th graders because they were highly involved in preparation for entrance examinations in their final years of middle and high schools^2^.

Before data collection, ethics approval for this study was granted by Northwest Minzu University. Research assistants contacted several public schools located in north mainland China. After obtaining permission from school principals, we administrated a passive consent procedure in this study (Hollmann and McNamara, 1999). Specifically, we asked head teachers in each classroom to send a message through parents’ Wechat groups to inform research purposes and participants’ rights of this study. If parents did not agree with this research activity, they should inform head teachers privately. In this context, the corresponding adolescents would not be allowed to participate in the following research program. The same procedure can be found in prior research of Chinese adolescents (e.g., Wang et al., 2019). Moreover, the confidentiality and anonymity nature of this study were strictly guaranteed in the process of data collection. During school hours, research assistants distributed the questionnaires to adolescents; meanwhile, adolescents were asked whether he or she would like to participate in this project. Adolescents who did not participate in this study were assigned other academic tasks by their head teachers. Following standardized instructions, adolescents were asked to complete a battery of questionnaires during a regular class hour in each classroom.

### Measures

#### Sociodemographic Characteristics

Adolescents were asked to answer a few questions at the beginning of this survey to indicate their sociodemographic backgrounds, such as sex, grade, date of birth, parental education, parental occupation, and family monthly income. Parental education, parental occupation, and family monthly income (for elaboration, see Cui and Lan, 2020) were first standardized and subsequently summed to yield a composite score, with higher scores indicating higher levels of family SES.

#### Bereaved Experience

In this study, we measured the bereaved experience of adolescents using a few questions adapted from Chan et al. (2012). To be specific, we asked adolescents the following questions: (1) if he or she had lost any close family members who resided together with them due to physical illness or accident (with a dichotomous answer format of yes or no) and (2) those who reported the death of close family members were asked additional questions to indicate their relationship with the deceased (i.e., fathers, mothers, siblings, or grandparents), the degree to which the loss of close family members severely influenced them (with a dichotomous answer format of yes or no), and age when their close family members passed away. The duration of losing close family members was calculated by the differences between adolescents’ date of birth and age when their close family members passed away.

#### Grit

Grit was measured by the 8-item Grit Scale (Duckworth and Quinn, 2009). This scale consists of two dimensions: perseverance and consistency, which has been validated in Chinese adolescents (Li et al., 2018). Item examples are “Setbacks do not discourage me (4 items; perseverance)” and “New ideas and projects sometimes distract me from previous ones (4 items; reversed-coded; consistency).” Participants were asked to rate all these items from 1 (*not like me at all*) to 5 (*very much like me*) on the Likert-type scale. All items related to each dimension were averaged to yield a score of perseverance and consistency, with a higher score indicating higher levels of perseverance and consistency. Previous studies have shown good internal consistency of this scale among Chinese adolescents (Lan, 2019; Lan, 2020; Lan and Moscardino, 2019; Ma et al., 2020). In the current study, Cronbach’s alphas were 0.82 and 0.80 for perseverance and consistency, respectively.

#### Parental Autonomy Support

Parental autonomy support was measured by the parental autonomy support questionnaire, which was developed by Wang et al. (2007). This questionnaire contains eight items (two dimensions: choice-making and opinion exchanges). One item example is “My parents allow me to make choices whenever possible (choice-making).” All these items were rated on a 5-point Likert-type scale ranging from 1 (*completely disagree*) to 5 (c*ompletely agree*). The mean score was yielded to represent the score of parental autonomy support, with a higher score indicating higher levels of perception of autonomy support from parents. Prior research has demonstrated good internal consistency of this scale in Chinese adolescents (Lan et al., 2019a; Lan and Wang, 2020a). In this study, Cronbach’s alpha was 0.86.

#### Teacher Autonomy Support

Teacher autonomy support was assessed using the Learning Climate Questionnaire (LCQ; Black and Deci, 2000), which consists of nine items. One item example is “I feel that my head teacher provides me choices and options.” Participants were asked to rate each item based on a Likert-type scale ranging from 1 (*strongly disagree*) to 5 (*strongly agree*). The mean score of all these items was obtained to represent the teacher autonomy support score, with higher values indicating higher levels of perception of autonomy support from head teachers. Prior research has shown good internal consistency of this scale in Chinese adolescents (Lan and Zhang, 2019). In this study, Cronbach’s alpha was 0.91.

#### Externalizing Problem Behavior

Externalizing problem behavior was measured by the subscales of the Youth Self-Report (YSR; Achenbach, 1991; Li et al., 2009), which is derived from the Child Behavior Checklist for the self-report purpose. The YSR has been validated in Chinese adolescents (Leung et al., 2006), showing adequate reliability and validity. The two subscales (i.e., rule-breaking behavior and aggressive behavior; 15 items) were adopted to represent externalizing problem behavior, as suggested by prior research (Achenbach and Rescorla, 2001; Rescorla et al., 2013). One item example is “I destroy my own things (aggressive behavior).” Adolescents were asked to assess each item based on a 4-point Likert-type scale ranging from 1 (*definitely does not apply to me*) to 4 (*definitely applies to me*). Following previous research (Li et al., 2009), the total score was calculated by averaging all items, with higher scores indicating more severe externalizing problem behavior. In this study, Cronbach’s alpha was 0.88.

#### Social Desirability

Social desirability was assessed by the Responding Desirably on Attitudes and Options Scale (RD-16; Schuessler et al., 1978), which has been adapted into the Chinese cultural contexts by Zhang et al. (2015). This scale consists of 16 items, and one item example is “I find that I can help others in many ways.” Participants were asked to rate each item based on a 7-point Likert scale ranging from 1 (*totally disagree*) to 7 (*totally agree*). The average score of all items was calculated, with a higher score indicating a higher level of social desirability. Prior research has established good internal consistency of this scale in Chinese adolescents (Yang et al., 2017). In this study, Cronbach’s alpha was 0.85.

### Data Analyses

Data analyses were performed in SPSS 21.0 (IBM Corporation, 2012), Mplus 7.0 (Muthén and Muthén, 2012), and Jamovi 1.1.9.0 (The Jamovi Project, 2020). Before data analyses, we used a two-step approach to addressing the impact of missing values in this study. The cut-off of high levels of missing data values per each participant was set at 20% in one of the questionnaires, as mentioned earlier in our battery (Lan et al., 2019b). In this study, 29 adolescents were identified and excluded in further analyses due to high levels of missing data. To examine the influence of remaining missing data (less than 20%), we conducted a Little’s Missing Completely at Random (MCAR) test (Little and Rubin, 1987). Results supported the MCAR assumption, χ^2^(35) = 35.35, and *p* = 0.45. Hence, the maximum likelihood estimates using the expectation-maximization (EM) algorithm were used to impute missing data (Enders, 2003).

Furthermore, as the present research relied extensively on self-report questionnaires, we performed Harman’s single-factor test to detect the impact of common method bias in this study. Harman’s single-factor test, as a *post hoc* procedure, is the widely used test to examine whether a single factor is accountable for variance in empirical studies (see a useful review by Tehseen et al., 2017). As recommended by prior research (Lan and Zhang, 2019), all items (study variables only) were loaded into an exploratory factor analysis to check whether one factor can explain the majority variance. The results indicated seven factors with initial eigenvalues greater than 1.00, and the first factor accounted for 21.51% of the variance. As compared with prior research using a similar methodology (Podsakoff et al., 2003; Wang et al., 2019), the common method bias is not a pervasive issue in this study.

Before addressing our research questions, we computed means, standard deviations, and zero-order correlations to have a preliminary overview of study variables. With regard to the first research question, we conducted an LPA using the maximum likelihood (ML) estimator in Mplus (Muthén and Muthén, 2012) to identify latent autonomy support profiles. Following prior research (e.g., Ma et al., 2019; Lan, 2020), we started with a one-profile model and systematically increased the number of profiles until a five-profile solution. Specifically, we evaluated each model using: information-theoretic methods [Akaike information criterion (AIC), Bayesian information criterion (BIC), and adjusted BIC], likelihood ratio statistical test methods [bootstrap likelihood ratio test (BLRT) and Lo–Mendell–Rubin adjusted likelihood ratio test (LMR-LRT)], and entropy-based criterion (see Nylund et al., 2007; Tein et al., 2013). An optimal model fit was selected in the context of lower AIC, BIC, and adjusted BIC values, higher entropy, a significant BLRT, and a significant LMR-LRT (Zhou et al., 2018). To ensure that these emerging profiles are differentiated, multivariate analysis of variance (MANOVA) and the follow-up *post hoc* test (i.e., Bonferroni correction) were conducted.

With regard to the second research question, a linear regression analysis was conducted to examine the direct and interactive associations of perseverance, consistency, and autonomy support profiles with externalizing problem behavior among family-bereaved adolescents^3^. In this analysis, age, sex, family SES, social desirability, the degree of possible trauma effect, and the duration of losing close family members were considered covariates. As illustrated by prior research (Lan and Radin, 2020), many researchers report a moderate association between perseverance and consistency among Chinese adolescents. Likewise, we aimed to incorporate many two-way interactions between perseverance/consistency and distinct autonomy support profiles. Given these empirical indications and our research aim, we performed two separate linear regression models, one for each dimension of grit. This was done to potentially decrease the multicollinearity issues, which may influence the variance of the coefficient estimates, and make the estimates very sensitive to minor changes in the model (Marsh et al., 2004; Shieh, 2011). Nevertheless, given the conceptual linkage between the two dimensions of grit (Duckworth et al., 2007; Duckworth and Quinn, 2009), we controlled another dimension when regarding one of the targeted dimensions as the predictor. Simple slope analysis was performed accordingly to detect the nature of the possible significant interactions in the regression model (Aiken et al., 1991; Lan and Wang, 2019). In these analyses, the level of significance was examined by *p*-value and 95% confidence intervals. The significance level was set at *p* < 0.05, and the confidence intervals should not contain zero (Lan and Moscardino, 2020).

## Results

### Descriptive Statistics

Means and standard deviations for study variables are presented in Table 1. As shown in Table 1, perseverance, consistency, parental autonomy support, and teacher autonomy support were significantly and negatively associated with externalizing problem behavior. In terms of covariates, adolescent boys reported higher levels of externalizing problem behavior than girls; social desirability was negatively related to externalizing problem behavior.

**TABLE 1:** Descriptive statistics and bivariate correlations of study variables for family-bereaved adolescents.

	*M*	*SD*	Range	1	2	3	4	5	6	7	8	9	10	11
(1) Perseverance	2.92	0.84	1–5	–										
(2) Consistency	3.45	0.82	1–5	0.23***	–									
(3) PAS	3.73	0.77	1–5	0.07*	0.26***	–								
(4) TAS	3.70	0.78	1–5	0.01	0.21***	0.30***	–							
(5) EPB	1.54	0.44	1–4	−0.21***	−0.17***	−0.28***	−0.20***	–						
(6) Age	15.74	1.53	13–18	–0.01	−0.12***	0.02	0.07*	–0.04	–					
(7) Sex^1^	–	–	1–2	–0.03	−0.15***	0.01	−0.07*	−0.11***	–0.01	–				
(8) SES	15.72	3.02	7–24	0.01	0.10**	0.04	–0.01	–0.04	0.06	–0.03	–			
(9) Degree	0.63	0.48	0–1	–0.01	0.01	–0.04	–0.03	0.05	−0.10**	0.03	0.02	–		
(10) Duration (years)	4.57	2.97	1–13	−0.09*	0.02	0.04	–0.01	0.02	0.07	0.01	–0.04	–0.02	–	
(11) Social desirability	5.33	0.79	1–7	0.08*	0.35***	0.42***	0.27***	−0.32***	0.01	0.06	–0.01	–0.02	–0.01	–

N = 763. ^1^coded as 1 = male, 2 = female. PAS, parental autonomy support; TAS, teacher autonomy support; EPB, externalizing problem behavior; and SES, socioeconomic status. The standardized score of SES ranged from −8.21 to 7.69. *p < 0.05, **p < 0.01, ***p < 0.001.

### Identification of Autonomy Support Profiles

An LPA was used to identify autonomy support profiles in family-bereaved adolescents. Results showed that the likelihood ratio statistical tests (i.e., LMR-LRT and BLRT values) were significant for the two-, three-, and five-profile solutions (see Table 2). A statistically significant likelihood ratio test suggests that the current model is preferred over a model with one less profile (Muthén and Muthén, 2012). However, the smallest profile in the five-profile solution accounted for 3.0% of participants only. As recommended by previous research (Merz and Roesch, 2011; Cui and Lan, 2020), solutions with small numbers of participants are considered as spurious profiles and cannot truly represent a unique latent subgroup. Moreover, the five-profile solution was not desirable for easing the interpretation of the results, as too many interaction terms between grit and emerging profiles would be created in the regression model. Among the remaining solutions, the three-profile option showed better fit statistics (i.e., lower levels of AIC, BIC, aBIC, and higher entropy). Given the considerations mentioned earlier, we proposed a three-profile solution as being optimal in this study.

**TABLE 2:** The goodness of fit indices for different latent autonomy support profiles.

	AIC	BIC	aBIC	Entropy	LMR-LRT	BLRT	Smallest profiles (%)
1-Profile	3570.92	3589.47	3576.76	–	–	–	–
2-Profile	3483.91	3516.37	3494.14	0.68	88.56***	93.01***	20.1%
**3-Profile**	**3453.02**	**3499.39**	**3467.64**	**0.75**	35.12*	36.88*	**4.5%**
4-Profile	3416.44	3476.73	3435.45	0.67	33.63	35.32	5.6%
5-Profile	3397.89	3472.09	3421.28	0.71	23.38*	24.55*	3.0%

N = 763. AIC, Akaike information criteria; BIC, Bayesian information criterion; aBIC, Adjusted Bayesian information; LMR-LRT, Lo–Mendell–Rubin adjusted likelihood ratio test; BLRT, Bootstrapped likelihood ratio test. *p < 0.05, ***p < 0.001. The optimal model is highlighted in bold type.

To further characterize these three profiles, a multivariate analysis of variance on parental autonomy support and teacher autonomy support was conducted. The results showed significant differences across the three profiles in the two selected indicators, except for the non-significant differences between profiles 1 and 2 in parental autonomy support. The mean differences in each indicator are reported in Table 3.

**TABLE 3:** Mean differences in study outcomes across three autonomy support profiles.

	1. HPHT		2. HPLT		3. LPLT				
	(*n* = 598)		(*n* = 34)		(*n* = 131)				
			
	*M*	*SD*	*M*	*SD*	*M*	*SD*	*F*	$\eta_{\text{p}}^{2}$	*Post hoc*
PAS	3.99	0.54	3.83	0.72	2.53	0.52	381.01***	0.50	1, 2 > 3
TAS	3.93	0.60	1.77	0.38	3.20	0.61	262.36***	0.40	1 > 3 > 2

N = 763. PAS, parental autonomy support; TAS, teacher autonomy support; HPHT, high parental autonomy support-high teacher autonomy support; HPLT, high parental autonomy support-low teacher autonomy support; and LPLT, low parental autonomy support-low teacher autonomy support. ***p < 0.001.

**FIGURE 1:**
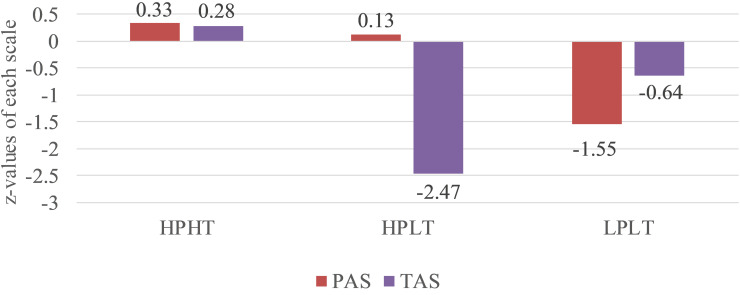
Three autonomy support profiles based on *z*-values of parental autonomy support and teacher autonomy support. *N* = 763. PAS, parental autonomy support; TAS, teacher autonomy support; HPHT, high parental autonomy support-high teacher autonomy support; HPLT, high parental autonomy support-low teacher autonomy support; and LPLT, low parental autonomy support-low teacher autonomy support.

Specifically, adolescents in the first profile (*n* = 598) reported the highest scores on both parental autonomy support and teacher autonomy support; thus, this profile was labeled “high parental autonomy support and high teacher autonomy support (HPHT)”; adolescents in the second profile (*n* = 34) reported moderate-to-high scores on parental autonomy support and the lowest scores on teacher autonomy support; hence, this profile was labeled “high parental autonomy support and low teacher autonomy support (HPLT)”; adolescents in the third profile (*n* = 131) reported the lowest scores on parental autonomy support and low-to-moderate scores on teacher autonomy support; therefore, this profile was labeled “low parental autonomy support and low teacher autonomy support (LPLT).” A graphical representation of these profiles is depicted in Figure 1.

### Associations of Perseverance, Consistency, and Autonomy Support Profiles With Externalizing Problem Behavior in Family-Bereaved Adolescents

Two separate linear regression models were used to examine the direct and interactive effect of perseverance/consistency and autonomy support profiles on externalizing problem behavior. In these analyses, we regarded the HPHT profile as the reference group and compared it with all the other profiles (i.e., HPLT and LPLT). As shown in Table 4, the model explained 14.9% of the variance on externalizing problem behavior, when perseverance was regarded as the predictor. Adolescents within the LPLT profile were more likely than those from the HPHT profile to report higher levels of externalizing problem behavior. Moreover, two-way interaction between perseverance and the profile contrast between HPHT and HPLT was significant. A simple slope analysis further examined the nature of this significant interaction.

**TABLE 4:** Regression analysis predicting externalizing problem behavior from perseverance and autonomy support profiles.

Variables	*B*	*SE*	95% CI		*t*	*p*
Perseverance (PE)	–0.01	0.03	–0.07	0.05	–0.26	0.79
HPLT vs. HPHT	0.11	0.07	–0.04	0.25	1.43	0.15
LPLT vs. HPHT	0.21	0.04	0.13	0.29	5.18	<0.001
Age	–0.01	0.01	–0.03	0.01	–1.46	0.14
Sex^1^	–0.12	0.03	–0.18	–0.06	–3.82	<0.001
SES	–0.01	0.01	–0.02	0.00	–1.37	0.17
Degree	0.04	0.03	–0.02	0.10	1.29	0.20
Duration (years)	–0.01	0.01	–0.02	0.00	–1.03	0.30
Social desirability	–0.17	0.05	–0.26	–0.07	–3.58	<0.001
Consistency	–0.07	0.02	–0.11	–0.03	–3.71	<0.001
PE × HPLT vs. HPHT	0.30	0.07	0.15	0.44	3.94	<0.001
PE × LPLT vs. HPHT	0.06	0.05	–0.04	0.15	1.21	0.23

*N = 763. ^1^coded as 1 = male, 2 = female. HPHT, high parental autonomy support-high teacher autonomy support; HPLT, high parental autonomy support-low teacher autonomy support; LPLT, low parental autonomy support-low teacher autonomy support; and SES, socioeconomic status.*

**FIGURE 2:**
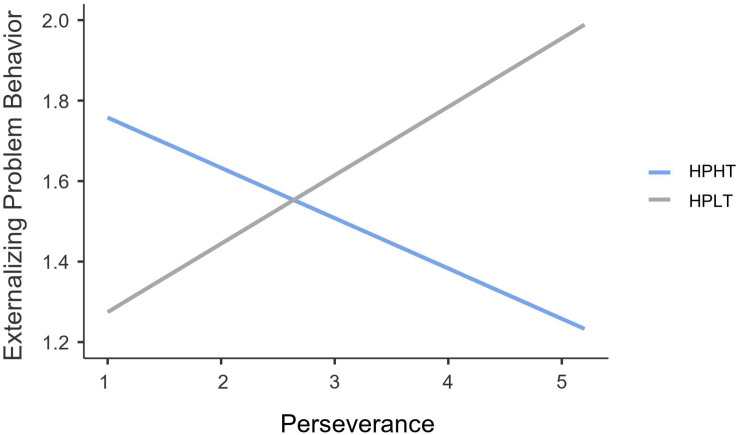
Interaction effect of perseverance and autonomy support profiles on externalizing problem behavior for Chinese family-bereaved adolescents. *N* = 763. HPHT, high parental autonomy support-high teacher autonomy support; HPLT, high parental autonomy support-low teacher autonomy support.

As shown in Figure 2, in terms of adolescents within the HPHT profile, perseverance was negatively and significantly related to externalizing problem behavior (*B* = −0.12, *SE* = 0.02, 95% CI = [−0.16, −0.08], *t* = −6.06, *p* < 0.001); however, this association was significantly positive for adolescents within the HPLT profile (*B* = 0.17, *SE* = 0.07, 95% CI = [0.02, 0.31], *t* = 2.34, *p* = 0.02).

When consistency was treated as the predictor, the results are shown in Table 5. As shown in Table 5, the model explained 18.7% of the variance in externalizing problem behavior. Adolescents within the LPLT and HPLT profiles were more likely than those from the HPHT profile to report higher levels of externalizing problem behavior. Moreover, two-way interaction between consistency and the profile contrast between HPHT and HPLT was significant.

**TABLE 5:** Regression analysis predicting externalizing problem behavior from consistency and autonomy support profiles.

Variables	*B*	*SE*	95% CI		*t*	*p*
Consistency (CO)	–0.02	0.03	–0.08	0.04	–0.68	0.50
HPHT vs. HPLT	0.16	0.07	0.01	0.30	2.16	0.03
LPLT vs. HPHT	0.21	0.04	0.12	0.29	4.90	<0.001
Age	–0.01	0.01	–0.03	0.01	–1.09	0.28
Sex^1^	–0.12	0.03	–0.18	–0.06	–3.85	<0.001
SES	–0.01	0.01	–0.02	0.00	–1.32	0.19
Degree	0.04	0.03	–0.02	0.10	1.23	0.22
Duration (years)	–0.01	0.01	–0.02	0.01	–0.98	0.33
Social desirability	–0.16	0.05	–0.25	–0.07	–3.45	<0.001
Perseverance	–0.10	0.02	–0.14	–0.07	–5.54	<0.001
CO × HPHT vs. HPLT	0.17	0.07	0.03	0.32	2.30	0.02
CO × LPLT vs. HPHT	–0.01	0.05	–0.11	0.09	–0.21	0.84

*N = 763. HPHT, high parental autonomy support-high teacher autonomy support; HPLT, high parental autonomy support-low teacher autonomy support; LPLT, low parental autonomy support-low teacher autonomy support; and SES, socioeconomic status. 1 coded as 1 = male, 2 = female.*

**FIGURE 3:**
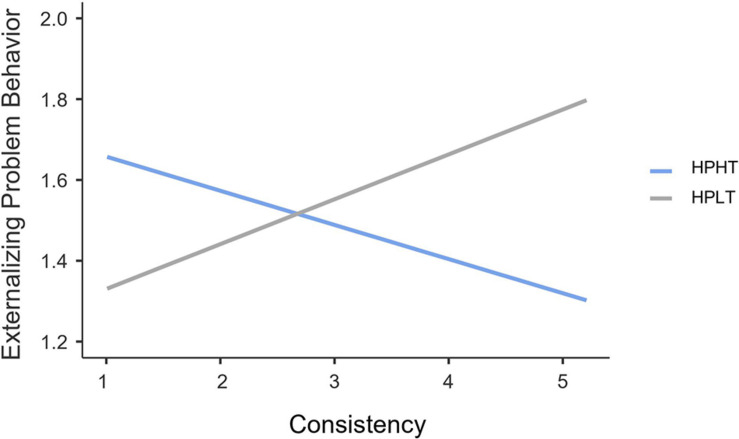
Interaction effect of consistency and autonomy support profiles on externalizing problem behavior for Chinese family-bereaved adolescents. *N* = 763. HPHT, high parental autonomy support-high teacher autonomy support; HPLT, high parental autonomy support-low teacher autonomy support.

As shown in Figure 3, regarding adolescents within the HPHT profile, consistency was negatively and significantly associated with externalizing problem behavior (*B* = −0.07, *SE* = 0.02, 95% CI = [−0.11, −0.03], *t* = −3.33, *p* < 0.001); in contrast, the corresponding association was not significant for those within the HPLT profile (*B* = 0.09, *SE* = 0.07, 95% CI = [−0.04, 0.23], *t* = 1.35, *p* = 0.17).

## Discussion

The current study aims to explore autonomy support profiles based on two centered figures (parents and head teachers) among family-bereaved adolescents. Likewise, we examine whether these emerging profiles may moderate the association between two dimensions of grit and externalizing problem behavior in Chinese family-bereaved adolescents. Although much research has documented that family-bereaved adolescents exhibit difficulties in terms of emotional and behavioral adaptation in Western cultural contexts, little is known about the corresponding situations in a collective setting and whether the cumulative effect of individual and environmental protective factors may ameliorate these potential difficulties. Attempting to broaden existing literature in this field, the current findings showed three autonomy support profiles: HPHT, HPLT, and LPLT. Moreover, perseverance and consistency were negatively related to externalizing problem behavior for family-bereaved adolescents within the HPHT profile. In contrast, the association between perseverance and externalizing problem behavior turned to be positive for family-bereaved adolescents within the HPLT profile.

The first purpose of this study was to explore autonomy support profiles among family-bereaved adolescents. The results from an LPA revealed three autonomy support profiles. The HPHT profile was characterized by high levels of autonomy support on all sources; the HPLT profile was described by the higher level of parental autonomy support and the lowest level of teacher autonomy support; and the LPLT profile was highlighted by low levels of autonomy support on all sources. These profiles further extend previous studies regarding perceived autonomy support profiles (Guay et al., 2013; Ratelle et al., 2013). Comparatively speaking, the HPHT profile represents the high proportion of family-bereaved adolescents. This may be because, in the past decades, China has witnessed dramatic modernization, urbanization, and related social changes. In a sense, Chinese parenting and teaching styles are potentially adjusted, with more endorsement of autonomy support. As documented by recent findings (e.g., Yu et al., 2016; Lan and Zhang, 2019), Chinese parents and teachers exhibit sizable autonomy support, which is highly related to adolescents’ positive psychosocial functions.

The second goal was to examine the direct and interactive associations of grit and autonomy support profiles with externalizing problem behavior. Results showed that perseverance and consistency were negatively related to externalizing problem behavior for family-bereaved adolescents within the HPHT profile. Such findings are following prior research (Blalock et al., 2015; Lan and Radin, 2020), suggesting that gritty individuals are capable of pushing through hardships, such as adverse life events. One possible explanation is that gritty adolescents may lessen the attention they devote to loss and grief by adjusting their focus to a long-term perspective, and satisfaction of autonomy support may accelerate adolescents’ integration of their bereavement experiences. Due to these salient personal and contextual strengths, family-bereaved adolescents are more capable of regulating their behaviors.

However, the association between perseverance and externalizing problem behavior turned to be positive for family-bereaved adolescents within the HPLT profile. It is noteworthy that the HPLT profile exhibits the lowest levels of teacher autonomy support and similar levels of parental autonomy support compared to those family-bereaved adolescents within the HPHT profile. One possible interpretation involves the crucial supporting role of teachers in Chinese adolescents. By promoting youths’ autonomous motivation, such as providing a social context in which adolescents feel that the learning process depends on themselves and their behavior is related to their interests, adolescents may function optimally concerning adaptive behavior (Núñez and León, 2015). Although parental autonomy support is critical in terms of adolescents’ psychosocial adjustment (e.g., Lan et al., 2019a), considering the large amount of time that adolescents spend at school during this period, it is possible that teacher autonomy support may have a more substantial effect than parental autonomy support on buffering externalizing problem behavior. Moreover, as family-bereaved adolescents attempt to seek comfort and support from their parents, they may find parents restricted in their autonomy support. This is because their parents may strive to manage their own emotions at first due to the loss of a close family member. In this context, teachers seem more available by providing opportunities for student autonomy. It is noteworthy that such a dark side of grit appears in the dimension of perseverance but not consistency. This is in accordance with prior research suggesting that being persistent in an unfavorable condition is linked to more impulsivity and aggression (Whiteside and Lynam, 2001; Anestis and Selby, 2015).

### Limitations and Possible Contributions

Although this study may significantly contribute to the literature by demonstrating protective factors against family-bereaved adolescents’ behavioral adaptation, the present findings should be interpreted in light of the following limitations. First, the current study relies on cross-sectional and correlational design. Although we attempt to control several potential confounding variables, this design has less power than a longitudinal design, when it comes to excluding time-invariants and unobserved individual differences (Lan and Wang, 2020b). For example, a retrospective report of family-bereaved experiences and other trauma-exposed factors may lead to an underestimation of the study associations. Moreover, this design cannot demonstrate the casualty of study correlations. To provide evidence about the temporal precedence between grit/autonomy support and externalizing problem behavior, additional studies are needed to clarify further the interaction between these variables on externalizing problem behavior over time.

Second, although the common method bias has been demonstrated to be minimal, and social desirability is statistically controlled in this study, the current findings may still be constrained by solely building on self-report questionnaires. For instance, prior research has indicated that externalizing problem behavior is better evaluated by external informants, such as teachers and parents (Leung et al., 2006). Moreover, parental autonomy support and teacher autonomy support are measured by the perception of autonomy support, but not the actual autonomy being nurtured by parents and head teachers. Prospective studies may consider asking parents and teachers to evaluate family-bereaved adolescents’ externalizing problem behavior, and to report their perception of autonomy support to family-bereaved adolescents.

Third, the current study focuses on a specific type of family bereavement, which has limited power to generalize into other bereaved populations. Future research should elaborate more on these associations by including a sample of parent-bereaved adolescents to gain a more comprehensive understanding of the correlations of externalizing problem behavior among family-bereaved adolescents. Within this perspective, a qualitative approach (e.g., structural interview) would be highly recommended, as parent-bereaved adolescents usually represent a very small group of adolescents in a general context. Relatedly, the current study fails to differentiate adolescents’ relationships with the deceased (siblings or grandparents), which could also affect the findings. Future research may consider unpacking this effect.

Fourth, the participant is recruited based on convenience sampling in this study, which may confront the sampling bias in its representativeness of study participants (Nielsen et al., 2017). For instance, in this study, all family-bereaved adolescents are recruited from urban public schools in north mainland China. Given the significant regional differences in China (rural vs. urban and north vs. south; Talhelm et al., 2014; Lan et al., 2019b), future studies should consider recruiting a nationally representative sample of family-bereaved adolescents. Based on random sampling and diverse samples, scientific conclusions may be more generalizable and meaningful.

Finally, although we have strived to address the protective factors against behavioral adaptation following bereavement in Chinese adolescents, the current research design delimits its applicability in other collectivist or individualist societies. Future studies should adopt a cross-cultural design (e.g., across different ethnicities or regions) to provide evidence of the cross-cultural similarities and differences in terms of the study associations.

## Conclusion

Despite these caveats, the present study may contribute to the literature in the following aspects. First, we use a person-centered approach to identify autonomy support profiles among family-bereaved adolescents, and the findings support the beneficial effect of autonomy support on family-bereaved adolescents’ behavioral adaptation in a collective cultural context. Second, the current study sheds light on the variability of the association between different dimensions of grit and problem behavior, demonstrating that this effect may depend largely on social support resources. Third, we show that the interplay between grit and favorable autonomy support from parents and teachers has a significant influence on buffering externalizing problem behavior. These significant findings may provide some solid foundations for designing targeted invention or prevention programs for Chinese family-bereaved adolescents.

## Data Availability Statement

The raw data supporting the conclusions of this article will be made available by the corresponding author, without undue reservation, to any qualified researcher.

## Ethics Statement

The studies involving human participants were reviewed and approved by Northwest Minzu University. Written informed consent to participate in this study was provided by the participants’ legal guardian/next of kin.

## Author Contributions

LF conceived and drafted the manuscript. XL performed the statistical analyses and critically revised the manuscript. All authors read and approved the final draft of the manuscript.

## Conflict of Interest

The authors declare that the research was conducted in the absence of any commercial or financial relationships that could be construed as a potential conflict of interest.

## Footnotes

1 Only a small group of adolescents reported the loss of one or both of their parents (*n* = 49), which may indicate that those adolescents may not a typical type of family-bereaved adolescents in the current sample. Statistically, we were interested in addressing our research questions based on a quantitative approach, and those small percentage of parent-bereaved adolescents prevented us from using such an approach. Moreover, from a theoretical point of view, losing a parent may present more significant detrimental effects on adolescents’ externalizing problem behavior compared with those who lost grandparents or siblings. In addition, given that parental autonomy support is one of the key study variables in this study, parent-bereaved adolescents or those whose parents divorced may potentially influence the quality of parental autonomy support, which obscures our study associations. Given these considerations, we decided to eliminate parent-bereaved adolescents and those adolescents whose parents divorced from the further course of analyses.

2 It is well-known that Chinese middle and high school students are confronted with great educational stress, particularly in the final years of their middle and high schools in China (Sun et al., 2013). This is because students in their last years of middle and high schools usually spend most of the time preparing for the entrance examinations, which are highly valued by Chinese parents and teachers (Tian et al., 2013). Moreover, due to salient academic stress, students in their final years of middle and high schools are prone to reporting distinct behavioral evaluations or identifying more elevated levels of problem behavior (as opposed to those not in their last years of middle and high schools), which may blur our study associations. Although excluding the students in their last years of middle and high schools may delimit the representativeness of research sample, it has been demonstrated a practical and reasonable solution, which has been applied in many empirical studies of Chinese adolescents (e.g., Tian et al., 2013; Lan and Zhang, 2019).

3 Given that the main objective of this study was to examine the moderating role of autonomy support profiles in the association between grit and externalizing problem behavior, a structural equation modeling (SEM) analysis would be ideal and valuable to assess our research questions. The general idea underlying SEM is the identification of a set of hypothetical underlying factors (latent) that linked to observed variables, and the identification of the speculative associations among these variables (MacCallum and Austin, 2000). Although adopting SEM may be theoretically desirable, it is not a practical method to address our research questions based on the following considerations. First, in this study, all measures have been developed or previously validated in the Chinese cultural context (see “Measures” section), thereby ensuring reliable estimates; likewise, we only regarded externalizing problem behavior as the dependent variable. Within these perspectives, SEM becomes statistically identical to the linear regression. Second, autonomy support profiles were regarded as nominal variables (non-continuous) in this study. When the indicators are categorical, we should modify the measurement model for continuous indicators, which complicates the model analysis and the interpretation of the results (for elaboration, see Skrondal and Rabe-Hesketh, 2014). Third, our targeted sample of this study was ‘atypical’ adolescents (i.e., family-bereaved adolescents), and the relatively small sample size of this study (*n* = 763) prevented us from using this approach, as it would have resulted in too many estimated parameters in relation to the number of study participants (Lan and Moscardino, 2019). As a note, these statistical considerations (i.e., linear regression instead of SEM) have been applied to many well-established empirical studies focusing on a specific group of adolescents (e.g., Ma et al., 2019; Lan and Wang, 2020b).
